# Five microRNAs in Serum Are Able to Differentiate Breast Cancer Patients From Healthy Individuals

**DOI:** 10.3389/fonc.2020.586268

**Published:** 2020-11-03

**Authors:** Andrea Feliciano, Lucila González, Yoelsis Garcia-Mayea, Cristina Mir, Mireia Artola, Nieves Barragán, Remedios Martín, Anna Altés, Josep Castellvi, Sergi Benavente, Santiago Ramón y Cajal, Martín Espinosa-Bravo, Javier Cortés, Isabel T. Rubio, Matilde E. LLeonart

**Affiliations:** ^1^Biomedical Research in Cancer Stem Cells Group, Vall d'Hebron Research Institute (VHIR), Universitat Autònoma de Barcelona, Barcelona, Spain; ^2^Primary Care Center CAP-Vallcarca-Sant Gervasi, Barcelona, Spain; ^3^Breast Pathology Unit, Vall d'Hebron University Hospital, Barcelona, Spain; ^4^Institute of Breast Cancer, Quiron Group, Barcelona, Spain; ^5^Vall d'Hebron Institute of Oncology (VHIO), Barcelona, Spain; ^6^Breast Surgical Oncology, University of Navarra Clinic, Madrid, Spain; ^7^Spanish Biomedical Research Network Center in Oncology, Madrid, Spain

**Keywords:** breast cancer, serum, microRNAs, prognosis, diagnosis

## Abstract

Breast cancer is the cancer with the most incidence and mortality in women. microRNAs are emerging as novel prognosis/diagnostic tools. Our aim was to identify a serum microRNA signature useful to predict cancer development. We focused on studying the expression levels of 30 microRNAs in the serum of 96 breast cancer patients *vs*. 92 control individuals. Bioinformatic studies provide a microRNA signature, designated as a predictor, based on the expression levels of five microRNAs. Then, we tested the predictor in a group of 60 randomly chosen women. Lastly, a proteomic study unveiled the overexpression and downregulation of proteins differently expressed in the serum of breast cancer patients *vs*. that of control individuals. Twenty-six microRNAs differentiate cancer tissue from healthy tissue, and 16 microRNAs differentiate the serum of cancer patients from that of the control group. The tissue expression of miR-99a, miR-497, miR-362, and miR-1274, and the serum levels of miR-141 correlated with patient survival. Moreover, the predictor consisting of miR-125b, miR-29c, miR-16, miR-1260, and miR-451 was able to differentiate breast cancer patients from controls. The predictor was validated in 20 new cases of breast cancer patients and tested in 60 volunteer women, assigning 11 out of 60 women to the cancer group. An association of low levels of miR-16 with a high content of CD44 protein in serum was found. Circulating microRNAs in serum can represent biomarkers for cancer prediction. Their clinical relevance and the potential use of the predictor here described are discussed.

## Introduction

Breast cancer is one of the most frequent carcinomas and the second leading cause of death in women (1). Specifically, in the United States and Europe, about 1 in 8 women (12.5%) will develop invasive breast cancer over the course of their life. Therefore, comprehensive research should be devoted to cancer prevention in order to scale down these numbers and reach higher life expectancy in affected patients, lower mortality rates, and decline socio-economical burdens due to the high cost of chemotherapeutical treatments.

Currently there is no precise model to estimate breast cancer risk. Most of the predictor models consider clinical factors, including the density of breast tissue, biopsy history, and several clinical parameters. However, such models are not informative at an individual level. Predictive tests (i.e., Oncotype DX, Prosigna, MammaPrint) based on the status of genetic and non-genetic factors in cancer tissue have proven their prognostic and predictive ability in a personalized way (2). Currently, the liquid biopsy is being used to establish the biomarkers that are able to predict or envisage a potential future cancer development risk (3).

microRNAs are key factors in oncogenesis because they contribute to the modulation of key oncogenic and tumor suppressor proteins. In particular, microRNA expression profiling can be used to classify human cancer (4). On the other hand, recent evidence suggests that microRNAs are very stable molecules in serum and that they have been established as biomarkers for some cancer types (5). Interestingly, the level of certain microRNAs in combination with known tumor markers (e.g., CEA or CA15-3) improves sensitivity to breast cancer detection (6). Thereby routine monitoring of circulating microRNAs can result in significant benefits for the prognosis, diagnosis, and breast cancer treatment (7).

Previously, we identified a molecular signature based on 35 microRNAs that vary significantly in normal tissue *vs*. cancer tissue in breast cancer patients (8). According to our previous work and literature search, we selected 30 cancer-related microRNAs that could be potentially detected in serum (miR-96, miR-451, miR-155, miR-195, miR-200c, miR-106b, miR-141, miR-21, miR-486, miR-16, miR-125b, miR-99a, miR-497, miR-191, miR-145, miR-100, miR-144, miR-382, miR-29c, miR-10b, miR-133a, miR-1260, miR-1274a, miR-1274b, miR-133b, miR-92, miR-376c, miR-411, miR-299, and miR-215) (8–11). In the present study, we compared the expression of 30 microRNAs in tumor *vs*. normal tissue and serum from 96 breast cancer patients (in comparison with control serum). Through statistical and bioinformatic studies, we determined a predictor, comprised by five microRNAs, that categorize an individual in the control group or breast cancer group. The potential benefit of this classifier and its validation for breast cancer prediction is discussed.

## Materials and Methods

### Patients and Controls

This study comprises 96 breast cancer patients. For each patient, we had samples of cancer tissue (CANtum), normal tissue (CANnorm), and serum (CANse). For comparison purposes, we had serum from 92 control individuals (CTLse). The method to select the control group established the following criteria: 20- to 80-year-old women, non-smokers, non-drinkers, no evidence of breast cancer in their family history, and healthy women that have had no cancer episodes in the past. For the validation study, we had additional serum from 20 breast cancer patients. Finally, for the test study, we had serum from 60 volunteer women where no selection criteria were applied. The pathological and clinical characteristics of the patients include the presence of estrogen receptor (ER), progesterone receptor (PR), Ki-67 expression, p53, tumor grade determined by tumor heterogeneity (low, medium, and high), tumor stage determined by the size of the tumor and its infiltrating capacity to neighboring local areas (T1b, T1c, or T2), subtype of breast cancer (molecular classification), presence of metastasis, disease-free survival, and overall survival. All patients included in the study were recruited from the Vall d'Hebron Hospital and selected for primary breast cancer. Patients were not treated with radio- or chemotherapy before sample collection. Control individuals were recruited from the Castilla-La Mancha Blood Bank and the Government of Catalonia Blood and Tissue Bank. Volunteer women came from the Primary Care Center (CAP-Vallcarca Sant Gervasi). The study was conducted in accordance with the instructions and requirements stated in the Declaration of Helsinki international standards for studies and approved by the Ethics Committee of Vall d'Hebron Hospital (CEIC). Informed consent was obtained from the patients to participate, analyze, and publish their data.

### Sample Collection

Serum was collected from each patient prior surgery. Hemolytic sera (representing 5%) were discarded from the study. Summing up, blood sample was obtained and centrifuged at 1,300 rpm for 10 min and the supernatant fraction (serum) was collected and stored at −80°C. The collection and pre-processing of the cancer samples vs. the healthy ones were treated with the same technical conditions. Normal and tumor tissue were collected from the surgery room and stored at −80°C before RNA extraction. Hematoxylin and eosin staining of the slides from frozen biopsies was validated histologically to ensure that the tissue area had an adequate tumor density (>80%). RNA was isolated with a MirVana kit (Ambion^®^ Life Technologies) according to the manufacturer's instructions. The RNA concentration from tissue was quantified using the Nanodrop-2000 UV-Vis Spectometer (Fisher Scientific) and its quality was determined by the Bioanalyser (RIN ratio> 8).

On the other hand, to verify that in RNA extractions from sera, there was enough RNA to analyze the 30 microRNAs considered in this study, each sample was amplified using RNU and cel-miR-39-3p probes individually using quantitative real-time qRT-PCR (data not shown).

### qRT-PCR

The reverse transcription was performed on 10 ng of RNA using specific primers for the 30 selected microRNAs, including endogenous control RNU6 (ID 00973) and the exogenous control cel-miR-39-3p (ID 000200) with the TaqMan commercial kit microRNA Reverse Transcription Kit (Applied Biosystems, Life Technologies, CA, USA) as described (8). The pre-amplification reaction was carried out on 5 μl of cDNA product using a pool containing the specific preamplification primers for each microRNA with the TaqMan^®^ PreAmp Master Mix 2 × solution (Applied Biosystems, Life Technologies, CA, USA). Reactions were performed in the Veriti™ Thermal Cycler Assays thermocycler (Applied Biosystems, Life Technologies, CA, USA). The study of each microRNA levels was conducted by triplicate. The references used for each microRNA are the following: mir-125b-5p (ID 000449), mir-99a-5p (ID 000435), mir-100-5p (ID 000437), mir-497-5p (ID 001043), mir-1274b (ID 002884), mir-106b-5p (ID 000442), mir-1260a (ID 002896), mir-141-3p (ID 000463), mir-96-5p (ID 000186), mir-21-5p (ID 000397), miR-1274a (ID 002883), mir-145-5p (ID 002278), mir-299-5p (000600), mir-376c-3p (ID 002122), mir-451a (ID 001141), mir-486-5p (ID 001278), cel-miR-39-3p (ID 000200), U6, miR-16-5p (ID 000391), mir-195-5p (ID 000494), mir-191-5p (ID 002299), mir-215-5p (ID 000518), mir-382-5p (ID 000572), mir-411-5p (ID 001610), mir-10b-5p (ID 002218), mir-155-5p (ID 002623), mir-200c-3p (ID 002300), mir-144-5p (ID 002148), mir-92a-3p (ID 000431), mir-133a-3p (ID 002246), mir-133b (ID 002247), mir29c-3p (ID 000587), and miR-362 (ID478058). Supplementary Tables 1, 2 show the raw qRT-PCR data for the indicated microRNAs in tissue and serum samples, respectively. Supplementary Tables 3, 4 show the qRT-PCR results for the indicated microRNAs in tissue and serum samples respectively upon normalization. The probes cel-miR-39-3p and RNU6 were used as internal controls, both to monitor the efficiency of RNA isolation and subsequent retrotranscription and to normalize possible variations between samples during RNA isolation. Although RNU6 is used as one of the most frequent endogenous controls to study profiling microRNA in cell and tissue samples, it is not a suitable endogenous control to study the expression of serum microRNAs (12). Therefore, in order to compare the data, results were normalized using the quantile method (using the normalized CtData function of the R package HTqRTPCR) including all patients per sample type.

### Proteomic Study

Serum from 70 breast cancer patients and 70 controls was studied at protein level. Each sample was depleted individually using the Pierce™ Abundant Protein Depletion Spin Columns kit (ref. 13434319, Thermo Scientific™) according to the manufacturer's instructions. This kit eliminates ~95% of 12 abundant proteins in serum (α1-Acid Glycoprotein, Fibrinogen, α1-Antitrypsin, Haptoglobin, α2-Macroglubulin, IgA, Albumin, IgG, Apolipoprotein A-I, IgM, Apolipoprotein A-II, and Transferrin), allowing the identification of other proteins in the samples. The quantitative study of proteins was performed through Tandem Mass Tag marking as previously described (13). Then, samples were grouped by pools (nine cancer pools and nine control pools) for sequencing. Each pool (80 μg of protein) was composed of equivalent amounts of seven samples of each type (cancer or control). Sequencing was performed by quantitative liquid chromatography tandem mass spectrometry using an LTQ-Orbitrap XL instrument as described above (14).

### Statistical Analysis

The study has been conducted using Leave-One-Out Cross Validation (LOOCV) as cross-validation technique, thus ensuring greater robustness in the results obtained (15).

Mann–Whitney *U*-test was used to identify microRNAs differently expressed between patients and controls. Benjamini–Hochberg's false discovery rate (FDR) method was used to correct for multiple testing. The analysis to select the differently expressed microRNAs has been based on the fitting of a linear model.

For the predictor, we considered that the best classification method was CART (Classification and Regression Trees) (16). The statistical analyses have been performed using ExpressionSuite (Life Technologies, CA, USA) (R version 3.5.1, copyright© 2018, Foundation for Statistical Computing, Vienna, Austria) and the libraries developed for microRNA-target analysis by the Bioconductor Project (www.bioconductor.org). Regarding the validation of the microRNA expression with the pathological characteristics of the patients, ANOVA and *t*-test methods were used (SPSS v9.3). A statistical analysis to determine differential proteins and peptides was performed using DanteR software (http://omics.pnl.gov/software/danter). *p* < 0.05 were considered significant.

## Results

### Tumor-Associated microRNAs in Breast Cancer

For the selection of the microRNAs studied here, they were selected: (a) the 17 most significantly deregulated microRNAs in breast cancer based on our previous work (miR-21, miR-96, miR-141, miR-1274a, miR-1260, miR-1274b, miR-106b, miR-299, miR-486, miR-376c, miR-497, miR-195, miR-100, miR-145, miR-99a, miR-451, and miR-125b) and (b) the potentially relevant microRNAs in the serum of breast cancer patients (miR-155, miR-200, miR-16, miR-191, miR-144, miR-382, miR-29c, miR-10b, miR-133a, miR-133b, miR-92, miR-411, and miR-215) (8–11, 17). The following microRNAs were studied in serum and cancer tissue in comparison with control individuals: miR-21, miR-96, miR-141, miR-1274a, miR-1260, miR-106b, miR-1274b, miR-299, miR-376c, miR-497, miR-195, miR-100, miR-145, miR-99a, miR-451, miR-125b miR-486, miR-16 (only serum), miR-191, miR-215, miR-382, miR-411, miR-106, miR-155, miR-200c, miR-144, miR-92a, miR-133a, miR-133b, miR-29c, and miR-362 (only tissue) (8).

**Figure 1:**
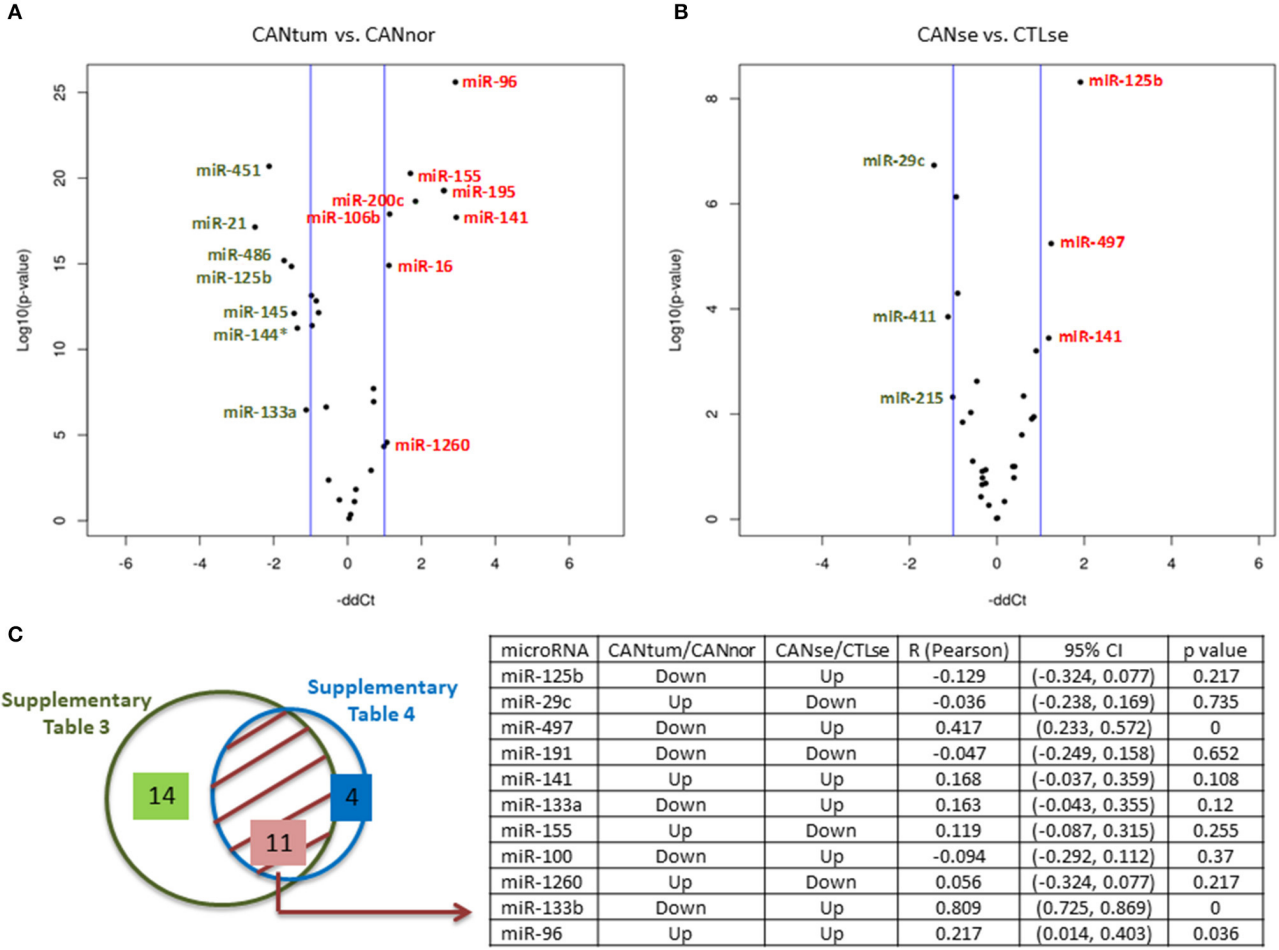
MicroRNAs expression in breast cancer. **(A)** Volcano plots indicating the top deregulated microRNAs when cancer tissue *vs*. normal tissue was compared (green, downregulated microRNAs; red, upregulated microRNAs). **(B)** Volcano plots indicating the top deregulated microRNAs when the serum of cancer patients was compared with a group of control serum. **(C)** Left side: Eleven commonly deregulated microRNAs when tissue and serum samples were compared. Right side: Table showing the potential association between the expression of 11 microRNAs in tissue and serum. It can be observed that three microRNAs (miR-497, miR-133b, and miR-96) have a statistically significant correlation coefficient (*R*) for a 95% confidence interval (CI) (*p* < 0.05). As indicated in the table, the microRNA values of the cancer tissue are relativized to normal tissue and the microRNA values of the cancer sera are relativized to the control sera. Up, upregulated; Down, downregulated.

Supplementary Table 5 shows the 26 microRNAs differently expressed when comparing tumor tissue with normal tissue in 96 breast cancer patients and 92 control individuals (*p* < 0.05). The volcano plot shows the most relevant microRNAs (Figure 1A) (*p* < 0.01). Supplementary Table 6 shows that 16 microRNAs (out of 30 initially selected) are significantly deregulated when comparing the serum from cancer patients *vs*. the serum from control individuals. The volcano plot shows the top significant microRNAs (Figure 1B). The miR-125b and RNU6 levels were validated by another approach based on the manual performance of the Assays-on-Demand Taqman Gene Expression Assays according to the procedure previously described (data not shown) (18). In order to check if the microRNAs expressed in the tumor reflect the same trend in the serum of breast cancer patients, we compared significant microRNAs in the tumor tissue and serum in all patients. Eleven out of 16 significant microRNAs were deregulated in both samples: tumor tissue of cancer biopsies and serum (Figure 1C, Supplementary Tables 5, 6). Three microRNAs, miR-191, miR-141, and miR-96, followed the same trend when the tumor and serum of cancer patients were compared (Figure 1C).

### Pathological and Clinic Characteristics of the Tumors

**Figure 2:**
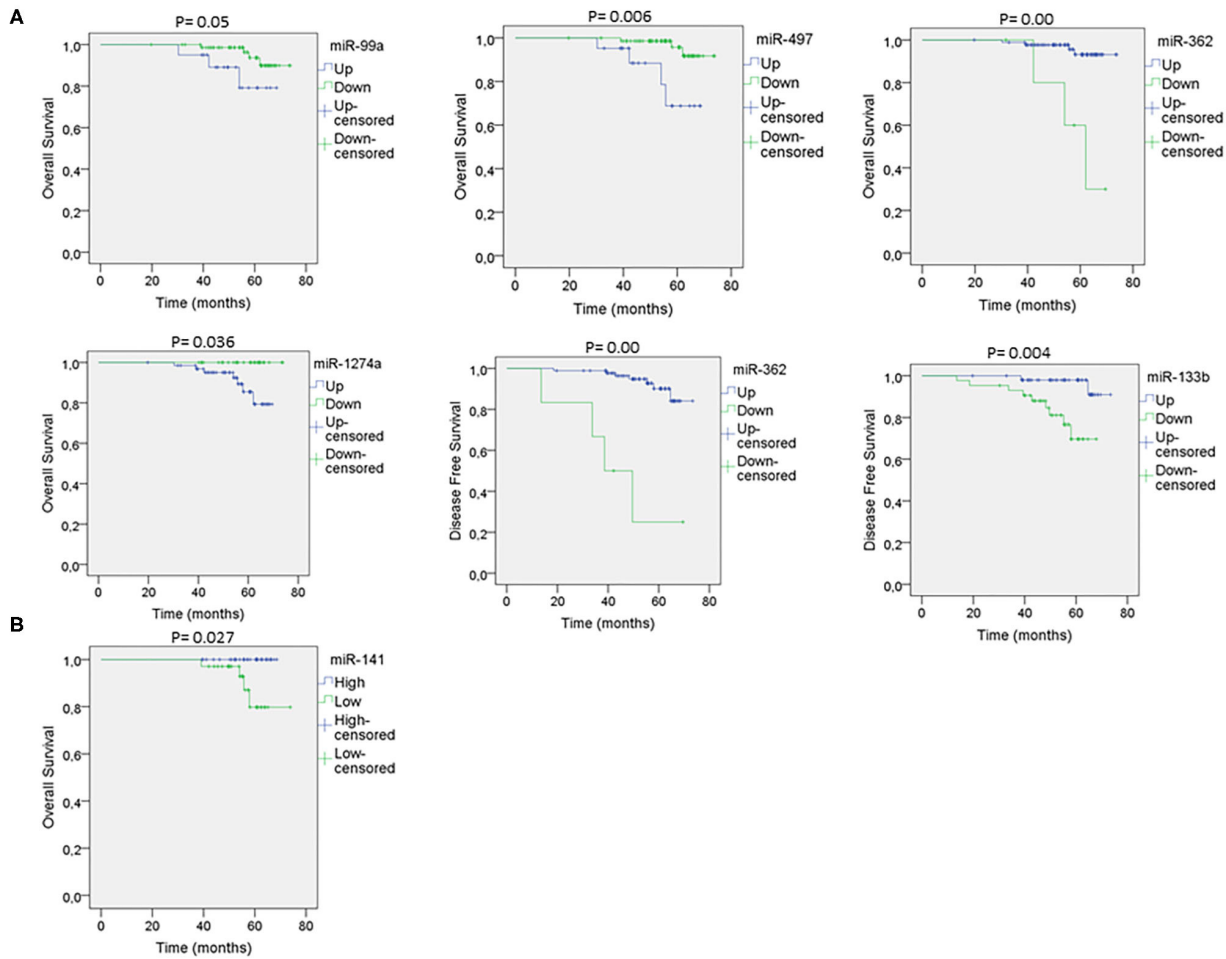
Prognosis-related microRNAs. **(A)** Kaplan–Meier curves showing that miR-99a, miR-497, miR-362, and miR-1274a levels in the tissue sample correlate with survival and/or disease-free survival. Up, upregulated; Down, downregulated. **(B)** Kaplan–Meier curve showing that miR-141 levels in the serum correlate with survival. Low, low expression; High, high expression.

The pathological characteristics of the patients are shown (Supplementary Table 7). Supplementary Figure 1 shows the serum microRNAs that correlate with tumor stage. Supplementary Figure 2 shows the tumor microRNAs that correlate with tumor grade. Supplementary Figure 3 shows the tumor microRNAs that correlate with tumor stage. We found that the expression of miR-99a, miR-497, miR-62, and miR-1274a correlated with overall survival (Figure 2A). In addition, miR-362 and miR-133b expression correlated with disease-free survival (Figure 2A). In addition, we found that high miR-141 expression in the serum of breast cancer patients correlated with better survival (Figure 2B). There is a lack of correlation regarding the studied microRNAs with the molecular classification of tumors (19).

### Construction of a Predictor

**Figure 3:**
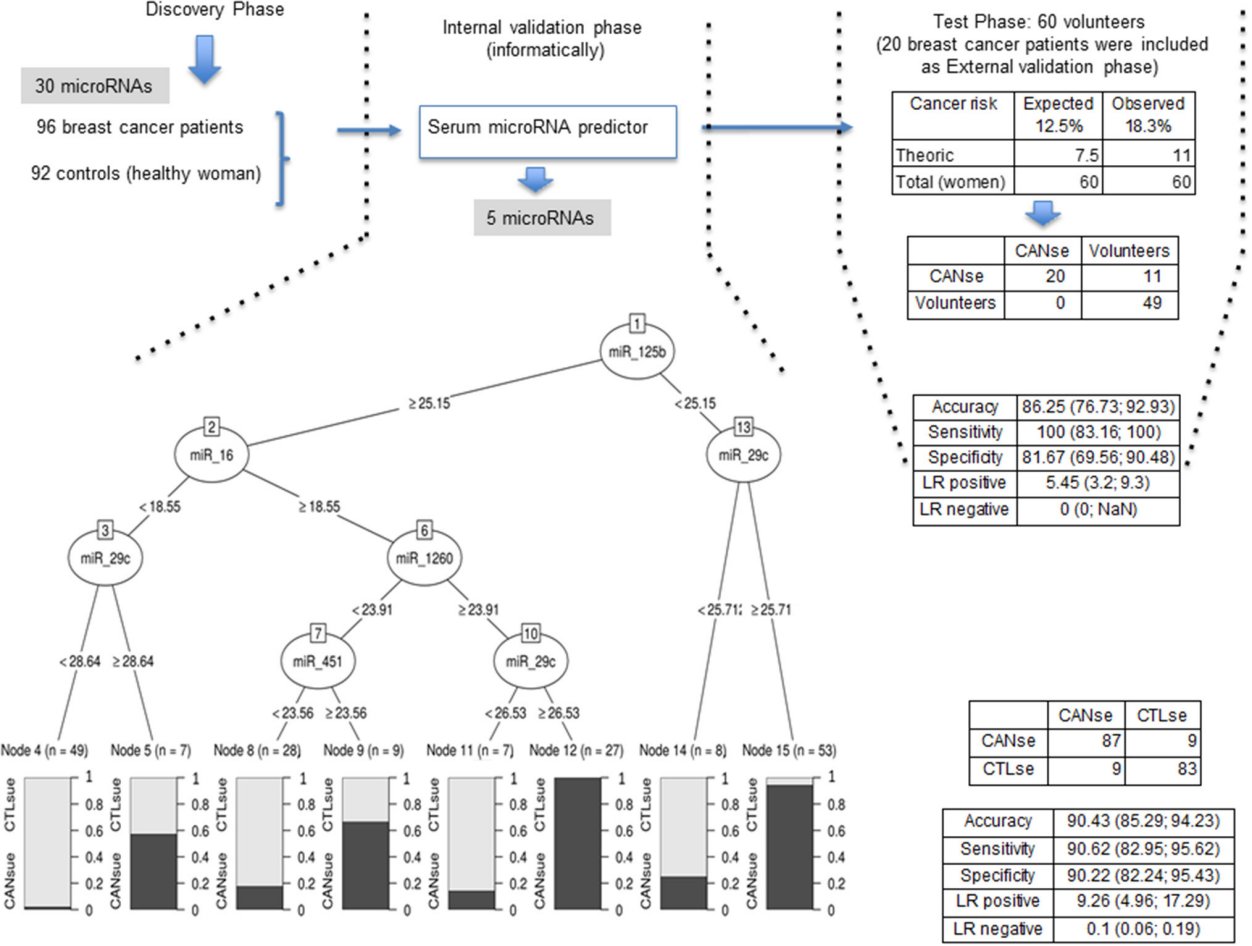
Flow chart of the analysis design in the present study. The expression change-based method pipeline is described (left). The interval validation phase provides 5 microRNAs revealed by the predictor (middle). The validation and test phase comprises 20 patients and apparently 60 healthy women (right). Indicated values of accuracy, sensitivity, and specificity are shown for each phase.

The experimental design of the study is summarized in Figure 3. In order to establish a microRNA signature designated here as predictor, statistical and bioinformatic studies were performed in the serum from 92 control women and 96 breast cancer patients. Accordingly, the minimal number of microRNAs able to predict whether a serum sample should be categorized as control or cancer was reduced to five: miR-125b, miR-29c, miR-16, miR-1260, and miR-451 (Figure 3). The proposed microRNA signature that derives exclusively from serum samples has the following percentages of accuracy, sensitivity, and specificity: 90.43, 90.62, and 90.22%, respectively (Figure 3). The internal classification error was 9.26%.

Later on, in an external validation phase, the predictor was used to verify the status of the serum from 20 additional cancer patients plus 60 serum samples from a group of volunteer women taken randomly to be tested by the predictor. Supplementary Table 8 shows the raw qRT-PCR data for the indicated microRNAs in serum samples. Supplementary Table 9 shows the qRT-PCR results for the indicated microRNAs in serum samples upon normalization. All serum samples were confirmed as cancer patients (Figure 3). Eleven out of 60 samples were classified as cancer patients (Figure 3). The percentages of accuracy, sensitivity, and specificity of this later study are 86.25, 100, and 81.67%, respectively (Figure 3). The internal classification error was 5.45%.

### Proteomic Study

**Figure 4:**
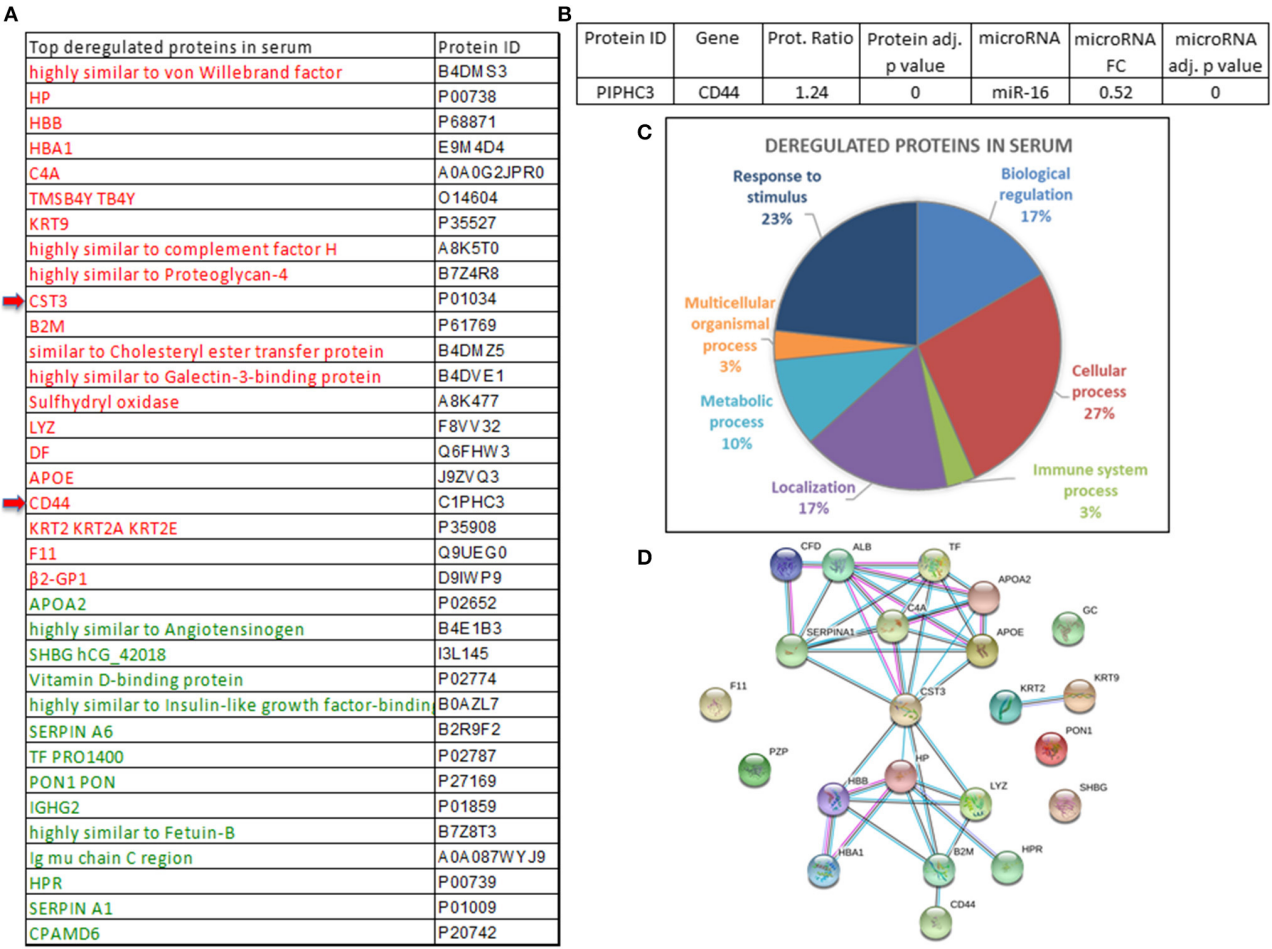
Proteins deregulated in the serum of breast cancer patients. **(A)** Proteins significantly deregulated in the serum pools of breast cancer patients *vs*. controls (upregulated proteins are indicated in red and those that are downregulated are indicated in green). **(B)** microRNAs and validated target proteins found in serum revealed miR-16 and CD44. FC, fold change. **(C)** Different molecular pathways involving significantly deregulated proteins. **(D)** Interactions of CST3 with other proteins which might have a relevant role in cancer.

A total of 110 significantly deregulated proteins were found when comparing the serum of cancer patients *vs*. the serum of healthy individuals (Supplementary Table 10). Thirty-five proteins were selected as the top differently expressed ones between cancer *vs*. normal serum using a fold change (FC) ratio above 1.2 or below 0.8 (Figure 4A). By using the multiMiR Bioconductor's package, microRNA–gene target interactions were explored (20). The search for validated targets was performed across miRecords, miRBase, and TarBase databases. A total of 3,947 validated unique target genes were found to the 16 microRNAs deregulated in serum (data not shown). CD44 protein (upregulated in the serum pools from breast cancer patients *vs*. the pools from the control group patients) was found in the list of the 3,947 validated targets. CD44 inversely correlates with miR-16 expression, which appears downregulated in the serum from cancer patients in comparison with controls (Supplementary Table 10, Figure 4B). The 35 proteins were classified accordingly to their involvement in different regulatory pathways (Figure 4C). Among them, CST3 (Cystatin C) seems to be involved in the modulation of different pathways (Figure 4D).

## Discussion

The final purpose of this research is to establish a microRNA signature associated with breast cancer to determine molecular evidence of cancer that will lead to future cancer development in serum samples. Firstly, we found 26 microRNAs significantly deregulated in the cancer *vs*. the healthy tissue from 96 breast cancer patients. Our results corroborate previous studies showing upregulation of miR-96, miR-200c, and miR-141, and downregulation of miR-145, miR-99a, and miR-125b in breast cancer tissue (8, 21–24). Secondly, we found that 16 out of 30 microRNAs were significantly deregulated in the serum of cancer patients *vs*. the serum of the control group. Interestingly, in serum of breast cancer patients, downregulation of miR-411, miR-376c, miR-16, and miR-155 (9, 17) and upregulation of miR-125b, miR-1260, and miR-96 had been previously described, confirming the validation of our results (9, 17, 21, 25). Some of these 16 microRNAs have been associated with breast cancer diagnosis including miR-125b, miR-191, miR-411, miR-155, and miR-215 (26, 27). In particular, 11 deregulated microRNAs were found in the serum and tissue of breast cancer patients (Figure 1C). Most of them are contrarily overexpressed among both types of samples, that is, although we found 11 deregulated microRNAs that are common to serum and tissue, their expression (either upregulated or downregulated) was inversely correlated when comparing serum and tissue. The fact that the expression of a specific microRNA in different sample types can have inverse implications in prognosis/diagnosis, has already been described as well as microRNA deregulation in the opposite direction when comparing their expression in serum *vs*. tissue (21, 28–30). For example, miR-125b, known to be downregulated in breast cancer tissue (8, 31), is upregulated in the bloodstream of breast cancer patients (21, 25, 32). Possible explanations include (i) extracellular and cellular microRNAs profiles differ, and freely circulating microRNA might not reflect their abundance in cancer cells (33); (ii) the total level of free microRNAs in the bloodstream might be masked by certain microRNAs present into exosomes (34).

In relation with the use of microRNAs as biomarkers, it has been suggested that the association of miR-99a in breast cancer tissue with survival differs depending on the molecular subtype (35). Our study corroborates the fact that high levels of miR-1274a are associated with worse prognosis and proposes two novel microRNAs associated with survival in breast cancer: miR-497 and miR-362 (36). Apart from miR-362, miR-133b correlates with disease-free survival, the latter already been described as a diagnostic marker in breast cancer (28). Interestingly, serum levels of miR-125b and miR-29c (the top 2 in order of significance; Figure 1C) were associated to tumor stage. Moreover, high levels of miR-141 in serum were correlated with better survival. Contrary to our results, Debel et al. found that miR-141 expression in serum was associated with shorter brain metastases (37).

Lastly, despite the growing interest in assessing predictive cancer models based on microRNA signatures, most of the reported studies need to be further evaluated in larger cohorts of breast cancer patients (21, 24, 38). In this study, we identified a predictor (based on the following microRNAs: miR-125b, miR-29c, miR-16, miR-1260, and miR-451), capable of differentiating the serum of breast cancer patients from that of control individuals with ~90% of accuracy, sensitivity, and specificity. The fact that the predictor model includes microRNAs less statistically significant such as miR-16 and miR-1260 than other more deregulated microRNAs is because the predictors work by combining different variables in a unique model to maximize discrimination between groups. The advantage of using a combination of variables is that predictive ability is obtained from the combination of this precise set of variables. That is, although some variable may show a small difference between groups, it may be the case that its contribution is different from other variables, so that including this variable in the model results in an increase of its global predictive capability. In a second phase, the predictor was validated and tested in 20 additional breast cancer cases plus 60 volunteer women, respectively. While the 20 patients were correctly categorized, the predictor included 11 out of 60 women into the cancer group. Although the theoretical breast cancer risk in the overall women population of Europe and United States is 12.5%, according to our predictor, we found a percentage of 18.3% women that will develop cancer in the future. This percentage (18.3%) represents an increase of ~1.5 over the expected values. A possible explanation of this high incidence could be the fact that, unlike the control group, this group of 60 women were not selected by any criteria; therefore, they could have a higher risk of developing breast cancer than the control group. It would be interesting to determine the health condition of those 60 women in the following 5–10 years with the purpose of establishing the validation of our predictor in the future.

On the other hand, differently expressed proteins in the serum of breast cancer patients *vs*. controls have been described (39). The deregulated proteins found in the pools of cancer *vs*. control serum samples—PEDF, IGKC, CD44, and CST3—have been previously reported (39–41). High levels of CD44 in serum are an independent prognosis indicator in primary breast cancer, since it correlates with overall survival and disease-free survival (42). Interestingly, we found that lower expression of miR-16 in the serum of cancer patients correlated with high expression of its CD44 target protein. Our results reinforce the potential relevance of CD44 as a potential marker of breast cancer as well as propose other proteins that might play key roles as biomarkers such as CST3, which needs to be extensively and individually studied in the serum of large series of patients (40).

Liquid biopsy (i.e., serum) is gaining importance in the clinical practice as novel biomarkers (i.e., microRNAs and proteins) are being considered to monitor healthy individuals. We hope that the results here reported open new avenues for future cancer prevention and diagnosis.

Overall, while much effort is being devoted to cancer predictive methods, it is not yet possible to detect cancer before the appearance of the first clinical symptoms. A molecular signature based on the detection in serum of five microRNAs capable of differentiating breast cancer patients from healthy individuals was found. The clinical application of the molecular signature herein described will be determined in large women's cohorts.

New microRNAs detected in serum and biopsy from breast cancer patients have been discovered. An association of low levels of miR-16 with a higher content of CD44 protein in serum was identified. This suggests the prognosis value of CD44 protein in serum as a potential marker of breast cancer. Collectively, our results support the fact that microRNA detection in serum can represent a viable predictive method applicable to breast cancer.

## Data Availability Statement

The information has been made public and accessible in the repository https://figshare.com/ with the updated information in the new document “Supplementary materials” provided.

## Ethics Statement

The studies involving human participants were reviewed and approved by Clinical Research Ethics Committee of Vall d'Hebron Hospital (CEIC). The patients/participants provided their written informed consent to participate in this study.

## Author Contributions

AF, LG, and ML conceived and designed the experiments. AF, LG, YG-M, and CM performed the experiments and designed the graphs. JCa and SB performed the statistical study. JCo, IR, NB, RM, and AA collected and organized the patients' information. AF, ME-B, SR, IR, JCa, and ML analyzed and interpreted the data. MA contributed to technical support. AF, YG-M, CM, NB, RM, AA, IR, SR, ME-B, JCo, and JCa contributed to scientific support. ML wrote the paper and conducted the study supervision. All authors read and approved the final manuscript.

## Conflict of Interest

JC holds Stock, patents and intellectual property of the company Medica Scientia Innovation Research (MedSIR). The remaining authors declare that the research was conducted in the absence of any commercial or financial relationships that could be construed as a potential conflict of interest.

## Abbreviations

CANnorm: normal tissue
CANtum: cancer tissue
CANse: cancer serum
Ct: cycle threshold
CTLse: control individual serum
dCt: Ct reference gene – Ct gene of interest
Down: downregulated
FC: Fold change
qRT-PCR: Quantitative Real-time PCR
RIN: RNA integrity number
Up: upregulated.
