# Supplementary material for: Five microRNAs in Serum Are Able to Differentiate Breast Cancer Patients From Healthy Individuals
Source: Front Oncol. 2020 Nov 3;10:586268. doi: 10.3389/fonc.2020.586268 (PMC7670964; doi:10.3389/fonc.2020.586268)
Supplement: Supplementary file 1 [file Data_Sheet_1.doc]

**I. Supplementary Tables:**

**Supplementary Table 1.**

<https://doi.org/10.6084/m9.figshare.12640415.v1>

**Supplementary Table 2.**

<https://doi.org/10.6084/m9.figshare.12640529.v1>

**Supplementary Table 3.**

<https://doi.org/10.6084/m9.figshare.12640547.v1>

**Supplementary Table 4.**

<https://doi.org/10.6084/m9.figshare.12640550.v1>

**Supplementary Table 5.** Normalized Ct data corresponding to the validation and test phases of the predictor in the serum of 20 breast cancer patients plus 60 test samples.

[https://www.frontiersin.org/articles/10.3389/fonc.2020.586268/full#supplementary-material](https://www.frontiersin.org/articles/10.3389/fonc.2020.586268/full#supplementary-material)

**Supplementary Table 6.** Proteomic study showing the deregulated proteins in the serum of cancer patients *versus* control individuals.

[https://www.frontiersin.org/articles/10.3389/fonc.2020.586268/full#supplementary-material](https://www.frontiersin.org/articles/10.3389/fonc.2020.586268/full#supplementary-material)

**Supplementary Table 7.**

<https://doi.org/10.6084/m9.figshare.12640565.v1>

**Supplementary Table 8.**

<https://doi.org/10.6084/m9.figshare.12640568.v1>

**Supplementary Table 9.**

<https://doi.org/10.6084/m9.figshare.12640574.v1>

**Supplementary Table 10.** Serum data (Ct) normalized (92 controls and 96 patients) for all studied microRNAs.

[https://www.frontiersin.org/articles/10.3389/fonc.2020.586268/full#supplementary-material](https://www.frontiersin.org/articles/10.3389/fonc.2020.586268/full#supplementary-material)

**II. Supplementary Figures:**

**Supplementary Figure 1.** Most relevant microRNAs expressed in the serum of cancer patients according to tumour stage.

<https://doi.org/10.6084/m9.figshare.13034852.v1>

**Supplementary Figure 2.** Most relevant microRNAs expressed in the tumour of cancer patients according to tumour grade.

<https://doi.org/10.6084/m9.figshare.13034996.v1>

**Supplementary Figure 3.** Most relevant microRNAs expressed in the tumour of cancer patients according to tumour stage.

<https://doi.org/10.6084/m9.figshare.13035005.v1>
