# Supplementary material for: Five microRNAs in Serum Are Able to Differentiate Breast Cancer Patients From Healthy Individuals
Source: Front Oncol. 2020 Nov 3;10:586268. doi: 10.3389/fonc.2020.586268 (PMC7670964; doi:10.3389/fonc.2020.586268)
Supplement: Supplementary file 2 [file Data_Sheet_1.PDF]

Supplementary Table 5. CANtum vs. CANnorm

microRNA	t test	p value	adj. p value	ddCt	FC (RQ)	CANtum	CANnorm
miR-96	-14,682	0	0	-2,919	7,563	26,411	29,282
miR-451	12,14	0	0	2,124	0,229	22,341	20,217
miR-155	-11,946	0	0	-1,7	3,25	19,775	21,475
miR-195	-11,39	0	0	-2,62	6,148	17,223	19,843
miR-200c	-11,196	0	0	-1,839	3,577	16,637	18,476
miR-106b	-10,855	0	0	-1,138	2,2	21,493	22,631
miR-141	-10,767	0	0	-2,942	7,683	19,96	22,902
miR-21	10,532	0	0	2,505	0,176	28,845	26,355
miR-486	9,619	0	0	1,717	0,304	21,969	20,252
miR-362	-9,488	0	0	-1,12	2,173	15,792	16,911
miR-125b	9,462	0	0	1,518	0,349	18,35	16,832
miR-99a	8,687	0	0	0,979	0,507	18,454	17,474
miR-497	8,544	0	0	0,848	0,556	22,416	21,568
miR-191	8,227	0	0	0,785	0,58	19,6	18,815
miR-145	8,22	0	0	1,447	0,367	16,069	14,632
miR-100	7,877	0	0	0,962	0,513	18,899	17,937
miR-144*	7,818	0	0	1,359	0,39	25,45	24,09
miR-382	-6,106	0	0	-0,702	1,627	24,09	24,792
miR-29c	-5,715	0	0	-0,707	1,632	25,385	26,092
miR-10b	5,557	0	0	0,579	0,67	21,02	20,45
miR-133a	5,466	0	0	1,121	0,46	24,678	23,589
miR-1260	-4,402	0	0	-1,063	2,089	17,548	18,61
miR-1274A	-4,259	0	0	-0,985	1,979	16,092	17,077
miR-1274B	-3,346	0,001	0,001	-0,636	1,553	12,577	13,213
miR-133b	2,929	0,004	0,005	0,514	0,7	25,743	25,225
miR-92a	-2,478	0,015	0,018	-0,225	1,168	21,429	21,654
