# Supplementary material for: Five microRNAs in Serum Are Able to Differentiate Breast Cancer Patients From Healthy Individuals
Source: Front Oncol. 2020 Nov 3;10:586268. doi: 10.3389/fonc.2020.586268 (PMC7670964; doi:10.3389/fonc.2020.586268)
Supplement: Supplementary file 3 [file Data_Sheet_2.PDF]

Supplementary Table 6. CANse vs. CTLse							
**microRNA**	**t test**	**p value**	**adj. p value**	**ddCt**	**FC (RQ)**	**CANse**	**CTLse**
miR-125b	-6,159	0	0	-1,913	3,765	24,893	26,844
miR-29c	5,429	0	0	1,44	0,369	28,224	26,805
miR-16	5,117	0	0	0,932	0,524	20,528	19,706
miR-497	-4,687	0	0	-1,243	2,366	25,512	26,342
miR-191	4,146	0	0	0,899	0,536	18,199	17,334
miR-411	3,911	0	0,001	1,119	0,46	29,409	28,304
miR-141	-3,652	0	0,002	-1,186	2,276	29,565	30,689
miR-133a	-3,478	0,001	0,003	-0,898	1,864	24,249	25,143
miR-155	3,08	0,002	0,008	0,461	0,727	24,356	23,907
miR-100	-2,877	0,005	0,014	-0,61	1,526	26,179	26,746
miR-215	2,864	0,005	0,014	1,012	0,496	28,461	27,451
miR-1260	2,626	0,009	0,025	0,598	0,661	24,449	23,822
miR-133b	-2,561	0,011	0,028	-0,846	1,797	25,702	26,331
miR-96	-2,563	0,012	0,028	-0,797	1,738	32,342	32,882
miR-376c	2,476	0,014	0,03	0,787	0,58	26,005	25,275
miR-99a	-2,263	0,025	0,05	-0,57	1,485	25,965	26,627
