# Supplementary material for: Five microRNAs in Serum Are Able to Differentiate Breast Cancer Patients From Healthy Individuals
Source: Front Oncol. 2020 Nov 3;10:586268. doi: 10.3389/fonc.2020.586268 (PMC7670964; doi:10.3389/fonc.2020.586268)
Supplement: Supplementary file 4 [file Data_Sheet_3.PDF]

Supplementary Table 10

Protein ID	Protein name	Gene name	Length (aa)
B4DMS3	cDNA FLJ59036, highly similar to von Willebrand factor	highly similar to von Willebrand factor	1104
P00738	Haptoglobin (Zonulin) [Cleaved into: Haptoglobin alpha chain; Haptoglobin beta chain]	HP	406
P68871	Hemoglobin subunit beta (Beta-globin) (Hemoglobin beta chain) [Cleaved into: LVV-hemorphin-7; Spinorphin]	HBB	147
E9M4D4	Hemoglobin alpha-1 globin chain (Fragment)	HBA1	100
A0A0G2JPR0	Complement C4-A	C4A	1744
O14604	Thymosin beta-4, Y-chromosomal	TMSB4Y TB4Y	44
P35527	Keratin, type I cytoskeletal 9 (Cytokeratin-9) (CK-9) (Keratin-9) (K9)	KRT9	623
A8K5T0	cDNA FLJ75416, highly similar to Homo sapiens complement factor H (CFH), mRNA	highly similar to complement factor H	1231
B7Z4R8	cDNA FLJ53364, highly similar to Proteoglycan-4 (Fragment)	highly similar to Proteoglycan-4	853
P01034	Cystatin-C (Cystatin-3) (Gamma-trace) (Neuroendocrine basic polypeptide) (Post-gamma-globulin)	CST3	146
P61769	Beta-2-microglobulin [Cleaved into: Beta-2-microglobulin form pI 5.3]	B2M	119
B4DMZ5	cDNA FLJ57826, moderately similar to Cholesteryl ester transfer protein	similar to Cholesteryl ester transfer protein	433
B4DVE1	cDNA FLJ53478, highly similar to Galectin-3-binding protein	highly similar to Galectin-3-binding protein	573
A8K477	Sulfhydryl oxidase (EC 1.8.3.2)	Sulfhydryl oxidase	604
F8VV32	Lysozyme C	LYZ	104
Q6FHW3	DF protein	DF	228
J9ZVQ3	Apolipoprotein E (Fragment)	APOE	238
C1PHC3	CD44 molecule (Fragment)	CD44	45
P35908	Keratin, type II cytoskeletal 2 epidermal (Cytokeratin-2e) (CK-2e) (Epithelial keratin-2e) (Keratin-2 epidermis) (Keratin-2e) (K2e) (Type-II keratin Kb2)	KRT2 KRT2A KRT2E	639
Q9UEG0	Coagulation factor XI (Fragment)	F11	126
D9IWP9	Beta-2-glycoprotein I (Fragment)	β2-GP1	326
Q19UH6	Truncated coagulation factor IX (Fragment)	F9	99
C9J6K0	Secreted phosphoprotein 24 (Fragment)	SPP2	131
A0A024R035	Complement component 9, isoform CRA_a	C9 hCG_37544	559
P20851	C4b-binding protein beta chain	C4BPB	252
P19652	Alpha-1-acid glycoprotein 2 (AGP 2) (Orosomucoid-2) (OMD 2)	ORM2 AGP2	201
P01011	Alpha-1-antichymotrypsin (ACT) (Cell growth-inhibiting gene 24/25 protein) (Serpin A3) [Cleaved into: Alpha-1-antichymotrypsin His-Pro-less]	SERPINA3 AACT GIG24 GIG25	423
B2R888	Monocyte differentiation antigen CD14 (Myeloid cell-specific leucine-rich glycoprotein)		375
P13645	Keratin, type I cytoskeletal 10 (Cytokeratin-10) (CK-10) (Keratin-10) (K10)	KRT10 KPP	584
P02763	Alpha-1-acid glycoprotein 1 (AGP 1) (Orosomucoid-1) (OMD 1)	ORM1 AGP1	201
P02743	Serum amyloid P-component (SAP) (9.5S alpha-1-glycoprotein) [Cleaved into: Serum amyloid P-component(1-203)]	APCS PTX2	223
Q6LAM1	Heavy chain of factor I (Fragment)		321
P02760	Protein AMBP [Cleaved into: Alpha-1-microglobulin (Protein HC) (Alpha-1 microglycoprotein) (Complex-forming glycoprotein heterogeneous in charge); Inter-alpha-trypsin inhibitor light chain (ITI-LC) (Bikunin) (EDC1) (HI-30) (Uronic-acid-rich	AMBP HCP ITIL	352
P13671	Complement component C6	C6	934
A0A024R962	HCG40889, isoform CRA_b	hCG_40889	1231
P51884	Lumican (Keratan sulfate proteoglycan lumican) (KSPG lumican)	LUM LDC SLRR2D	338
Q9UNU2	Complement protein C4B frameshift mutant (Fragment)	C4B	354
B4E1D8	cDNA FLJ51597, highly similar to C4b-binding protein alpha chain		536
Q06033	Inter-alpha-trypsin inhibitor heavy chain H3 (ITI heavy chain H3) (ITI-HC3) (Inter-alpha-inhibitor heavy chain 3) (Serum-derived hyaluronan-associated protein) (SHAP)	ITIH3	890
P22792	Carboxypeptidase N subunit 2 (Carboxypeptidase N 83 kDa chain) (Carboxypeptidase N large subunit) (Carboxypeptidase N polypeptide 2) (Carboxypeptidase N regulatory subunit)	CPN2 ACBP	545
B2R8I2	cDNA, FLJ93914, highly similar to Homo sapiens histidine-rich glycoprotein (HRG), mRNA		525
B4E1Z4	cDNA FLJ55673, highly similar to Complement factor B (EC 3.4.21.47)		1266
Q16519	Protein S (Fragment)	PROS1	650
H6VRF8	Keratin 1	KRT1	644
P27918	Properdin (Complement factor P)	CFP PFC	469
A0A087X232	Complement C1s subcomponent	C1S	682
A8K5J8	cDNA FLJ75066, highly similar to Homo sapiens complement component 1, r subcomponent (C1R), mRNA		705
P49908	Selenoprotein P (SeP)	SELENOP SELP SEPP1	381
P10643	Complement component C7	C7	843
B4DPP8	cDNA FLJ53075, highly similar to Kininogen-1		415
A0A096LPE2	SAA2-SAA4 readthrough	SAA2-SAA4	208
B7Z549	cDNA FLJ56821, highly similar to Inter-alpha-trypsin inhibitor heavy chain H1		677
B7Z550	Complement component 8, beta polypeptide, isoform CRA_b (cDNA FLJ59731, highly similar to Complement component C8 beta chain)	C8B hCG_23458	529
Q5VY30	Retinol binding protein 4, plasma, isoform CRA_b (Retinol-binding protein 4)	RBP4 hCG_37964	199
P07360	Complement component C8 gamma chain	C8G	202
P06681	Complement C2 (EC 3.4.21.43) (C3/C5 convertase) [Cleaved into: Complement C2b fragment; Complement C2a fragment]	C2	752

Protein ID	Protein name	Gene name	Length (aa)
P25311	Zinc-alpha-2-glycoprotein (Zn-alpha-2-GP) (Zn-alpha-2-glycoprotein)	AZGP1 ZAG ZNGP1	298
P36955	Pigment epithelium-derived factor (PEDF) (Cell proliferation-inducing gene 35 protein) (EPC-1) (Serpin F1)	SERPINF1 PEDF PIG35	418
P04217	Alpha-1B-glycoprotein (Alpha-1-B glycoprotein)	A1BG	495
P01024	Complement C3 (C3 and PZP-like alpha-2-macroglobulin domain-containing protein 1) [Cleaved into: Complement C3 beta chain; C3-beta-c (C3bc); Complement C3 alpha chain; C3a anaphylatoxin; Acylation stimulating protein (ASP) (C3ade	C3 CPAMD1	1663
C0JYY2	Apolipoprotein B (Including Ag(X) antigen) (Apolipoprotein B (Including Ag(X) antigen), isoform CRA_a)	APOB hCG_20898	4563
O75636	Ficolin-3 (Collagen/fibrinogen domain-containing lectin 3 p35) (Collagen/fibrinogen domain-containing protein 3) (Hakata antigen)	FCN3 FCNH HAKA1	299
P07357	Complement component C8 alpha chain (Complement component 8 subunit alpha)	C8A	584
P01031	Complement C5 (C3 and PZP-like alpha-2-macroglobulin domain-containing protein 4) [Cleaved into: Complement C5 beta chain; Complement C5 alpha chain; C5a anaphylatoxin; Complement C5 alpha' chain]	C5 CPAMD4	1676
P10909	Clusterin (Aging-associated gene 4 protein) (Apolipoprotein J) (Apo-J) (Complement cytolysis inhibitor) (CLI) (Complement-associated protein SP-40,40) (Ku70-binding protein 1) (NA1/NA2) (Testosterone-repressed prostate message 2) (TRP	CLU APOJ CLI KUB1 AAG4	449
P43652	Afamin (Alpha-albumin) (Alpha-Alb)	AFM ALB2 ALBA	599
Q5T985	Inter-alpha-trypsin inhibitor heavy chain H2	ITIH2	935
A0A024R9Q1	Thrombospondin 1, isoform CRA_a	THBS1 hCG_1787130	1170
O95445	Apolipoprotein M (Apo-M) (ApoM) (Protein G3a)	APOM G3A NG20 HSPC336	188
P00747	Plasminogen (EC 3.4.21.7) [Cleaved into: Plasmin heavy chain A; Activation peptide; Angiostatin; Plasmin heavy chain A, short form; Plasmin light chain B]	PLG	810
K7ER74	APOC4-APOC2 readthrough (NMD candidate)	APOC4-APOC2	178
A8K5A4	cDNA FLJ76826, highly similar to Homo sapiens ceruloplasmin (ferroxidase) (CP), mRNA		1065
P43251	Biotinidase (Biotinase) (EC 3.5.1.12)	BTD	543
B4DNT5	cDNA FLJ60316, highly similar to Apolipoprotein-L1		277
P01834	Immunoglobulin kappa constant (Ig kappa chain C region) (Ig kappa chain C region AG) (Ig kappa chain C region CUM) (Ig kappa chain C region EU) (Ig kappa chain C region OU) (Ig kappa chain C region ROY) (Ig kappa chain C region TI)	IGKC	107
P01008	Antithrombin-III (ATIII) (Serpin C1)	SERPINC1 AT3 PRO0309	464
P05546	Heparin cofactor 2 (Heparin cofactor II) (HC-II) (Protease inhibitor leuserpin-2) (HLS2) (Serpin D1)	SERPIND1 HCF2	499
A0A087X1J7	Glutathione peroxidase	GPX3	225
P08697	Alpha-2-antiplasmin (Alpha-2-AP) (Alpha-2-plasmin inhibitor) (Alpha-2-PI) (Serpin F2)	SERPINF2 AAP PLI	491
P02647	Apolipoprotein A-I (Apo-AI) (ApoA-I) (Apolipoprotein A1) [Cleaved into: Proapolipoprotein A-I (ProapoA-I); Truncated apolipoprotein A-I (Apolipoprotein A-I(1-242))]	APOA1	267
P04004	Vitronectin (VN) (S-protein) (Serum-spreading factor) (V75) [Cleaved into: Vitronectin V65 subunit; Vitronectin V10 subunit; Somatomedin-B]	VTN	478
P06727	Apolipoprotein A-IV (Apo-AIV) (ApoA-IV) (Apolipoprotein A4)	APOA4	396
B2R815	cDNA, FLJ93695, highly similar to Homo sapiens serpin peptidase inhibitor, clade A (alpha-1 antiproteinase, antitrypsin), member 4 (SERPINA4), mRNA		427
H0Y485	Insulin-like growth factor-binding protein 3 (Fragment)	IGFBP3	143
P02671	Fibrinogen alpha chain [Cleaved into: Fibrinopeptide A; Fibrinogen alpha chain]	FGA	866
P04196	Histidine-rich glycoprotein (Histidine-proline-rich glycoprotein) (HPRG)	HRG	525
B7Z8B6	cDNA FLJ54395, highly similar to Inter-alpha-trypsin inhibitor heavy chain H1		623
I3L0A1	Cysteine-rich secretory protein 3 (Fragment)	CRISP3	191
A5YAK2	Apolipoprotein C-IV	APOC4	127
A0M8Q9	C1 segment protein (HCG2040025) (Fragment)	C1 segment hCG_2040025	105
A0A1B1CYC5	Vitamin D binding protein (Fragment)	Gc	34
Q8N118	Cytochrome P450 4X1 (EC 1.14.14.1) (CYPIVX1)	CYP4X1 UNQ1929/PRO4404	509
B4E180	cDNA FLJ61201, highly similar to Beta-Ala-His dipeptidase (EC 3.4.13.20)		493
Q86TT1	Full-length cDNA clone CS0DD006YL02 of Neuroblastoma of Homo sapiens (human)		375
A0A0R7FJH5	Coagulation factor XII	F12	615
P02652	Apolipoprotein A-II (Apo-AII) (ApoA-II) (Apolipoprotein A2) [Cleaved into: Proapolipoprotein A-II (ProapoA-II); Truncated apolipoprotein A-II (Apolipoprotein A-II(1-76))]	APOA2	100
B4E1B3	cDNA FLJ53950, highly similar to Angiotensinogen	highly similar to Angiotensinogen	466
I3L145	Sex hormone-binding globulin (Sex hormone-binding globulin, isoform CRA_a)	SHBG hCG_42018	344
P02774	Vitamin D-binding protein (DBP) (VDB) (Gc protein-derived macrophage activating factor) (Gc-MAF) (GcMAF) (Gc-globulin) (Group-specific component) (Gc) (Vitamin D-binding protein-macrophage activating factor) (DBP-maf)	Vitamin D-binding protein	474
B0AZL7	cDNA, FLJ79457, highly similar to Insulin-like growth factor-binding proteincomplex acid labile chain	highly similar to Insulin-like growth factor-binding	605
B2R9F2	cDNA, FLJ94361, highly similar to Homo sapiens serine (or cysteine) proteinase inhibitor, clade A(alpha-1 antiproteinase, antitrypsin), member 6 (SERPINA6), mRNA	SERPIN A6	405
P02787	Serotransferrin (Transferrin) (Beta-1 metal-binding globulin) (Siderophilin)	TF PRO1400	698
P27169	Serum paraoxonase/arylesterase 1 (PON 1) (EC 3.1.1.2) (EC 3.1.1.81) (EC 3.1.8.1) (Aromatic esterase 1) (A-esterase 1) (K-45) (Serum aryldialkylphosphatase 1)	PON1 PON	355
P01859	Immunoglobulin heavy constant gamma 2 (Ig gamma-2 chain C region) (Ig gamma-2 chain C region DOT) (Ig gamma-2 chain C region TIL) (Ig gamma-2 chain C region ZIE)	IGHG2	326
B7Z8T3	cDNA FLJ50352, highly similar to Fetuin-B	highly similar to Fetuin-B	234
A0A087WYJ9	Deleted.	Ig mu chain C region	
P00739	Haptoglobin-related protein	HPR	348
P01009	Alpha-1-antitrypsin (Alpha-1 protease inhibitor) (Alpha-1-antiproteinase) (Serpin A1) [Cleaved into: Short peptide from AAT (SPAAT)]	SERPIN A1	418
P20742	Pregnancy zone protein (C3 and PZP-like alpha-2-macroglobulin domain-containing protein 6)	CPAMD6	1482
